# Supplementary material for: Anatomy Nights: An international public engagement event increases audience knowledge of brain anatomy
Source: PLoS One. 2022 Jun 9;17(6):e0267550. doi: 10.1371/journal.pone.0267550 (PMC9182231; doi:10.1371/journal.pone.0267550)
Supplement: S1 File — The double-sided test that each participant completed before and after the event. (PDF) [file pone.0267550.s001.pdf]

Anatomy Nights – Brains (Pre)

By completing the survey and returning it to a volunteer or placing it into the submission box you consent to participate in this study and for your answers here to be used freely for both instruction and research.

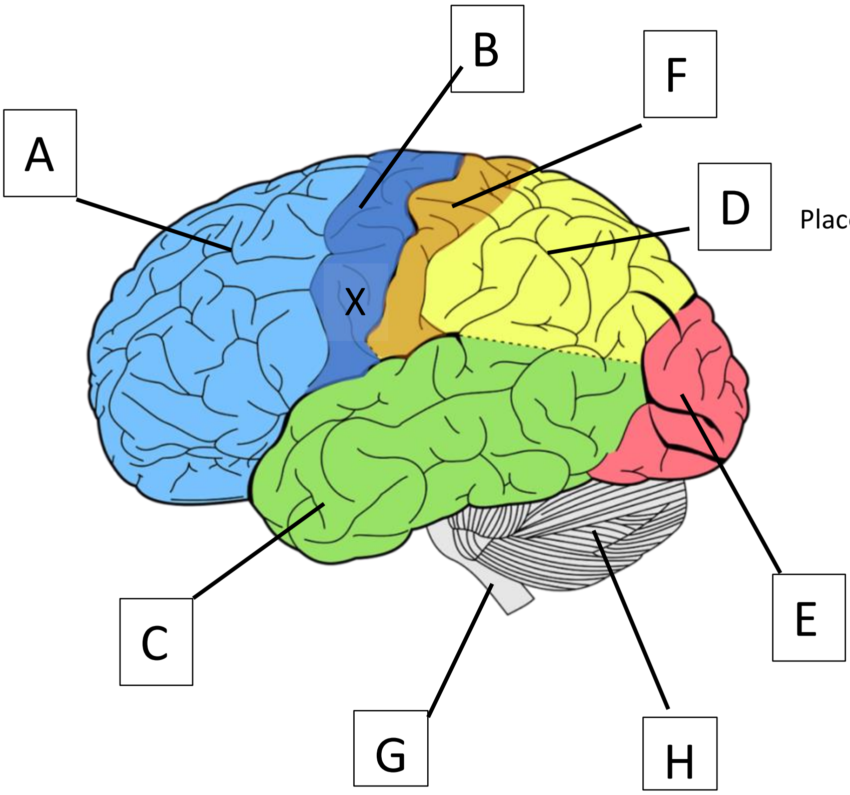

Place the letter of the structure after its description

Can you identify the following:-

The brain stem. …………….

The cerebellum. ……………..

The frontal lobe …………….

The occipital lobe …………….

The parietal lobe. …………….

The primary motor cortex ………

The primary sensory cortex ……..

Please tell us about yourself

(circle as appropriate)

1. Age……….
2. Gender: Man/ Woman/Gender Diverse/prefer not to say
3. Qualifications held School / Undergrad /Postgrad /None
4. Work in healthcare Y/N

If a stroke affected the area marked by X we would expect to see loss of function in

Right upper limbs

Left upper limbs

Right lower limbs

Left lower limbs

Anatomy Nights – Brains (Post)

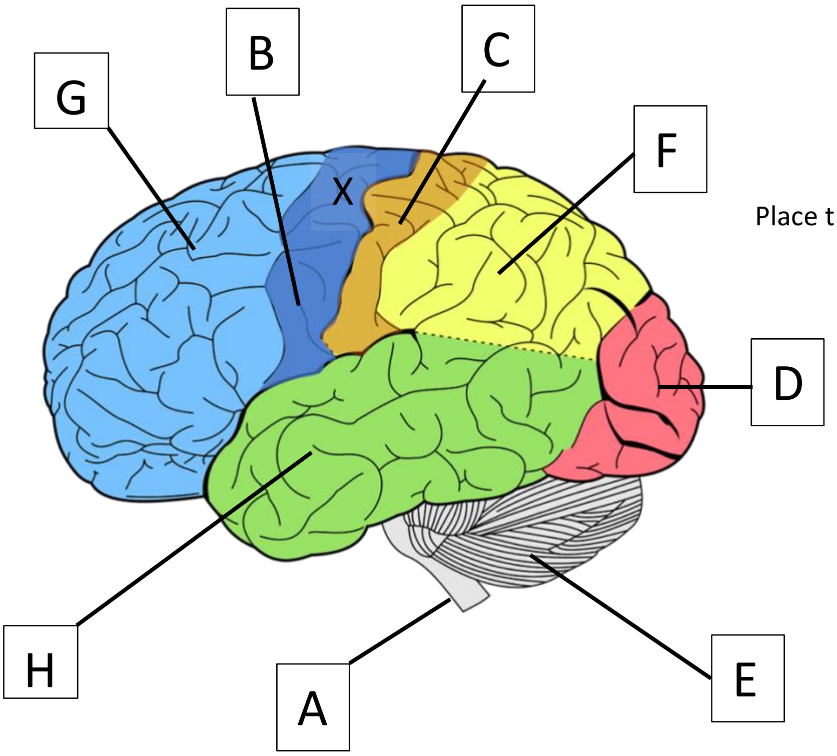

Place the letter of the structure after its description

Can you identify the following:-

The brain stem. …………….

The cerebellum. ……………..

The frontal lobe …………….

The occipital lobe …………….

The parietal lobe. …………….

The primary motor cortex ………

The primary sensory cortex ……..

If a stroke affected the area marked by X we would expect to see loss of function in

Right upper limbs

Left upper limbs

Right lower limbs

Left lower limbs

Thank you for taking part.

Find out more about Anatomy Nights and join the mailing list at **anatomynights.com** or follow us on Twitter @AnatomyNight
