# Supplementary material for: Anatomy Nights: An international public engagement event increases audience knowledge of brain anatomy
Source: PLoS One. 2022 Jun 9;17(6):e0267550. doi: 10.1371/journal.pone.0267550 (PMC9182231; doi:10.1371/journal.pone.0267550)
Supplement: S1 Table — (DOCX) [file pone.0267550.s003.docx]

**Supporting information 2**

**Estimates from Generalized Linear Mixed Models for effects of Academic Qualification, Employment in Healthcare, and Location on test performance. Results presented on the logit (for means) or log odds ratio (for contrasts) scales, averaged over effect of test timing (pre-, post-). 95% CI’s for contrasts are not adjusted for multiple comparisons.**

| **Academic Qualification** | | | | |
| --- | --- | --- | --- | --- |
| **Level** | **Estimated marginal mean** | **SE** | **95% CI** | |
| Post | 1.35 | 0.147 | 1.060, 1.64 | |
| School | 1.37 | 0.304 | 0.767, 1.97 | |
| Under | 1.23 | 0.164 | 0.907, 1.55 | |
| **Contrast** | **Log odds ratio** | **SE** | **df** | **95% CI** |
| post - school | -0.0182 | 0.335 | 174 | -0.680, 0.643 |
| post - under | 0.1195 | 0.215 | 174 | -0.305, 0.544 |
| school - under | 0.1377 | 0.343 | 174 | -0.539, 0.815 |
| **Work in Healthcare** | | | | |
| **Level** | **Estimated marginal mean** | **SE** | **95% CI** | |
| No | 1.23 | 0.121 | 0.988, 1.46 | |
| Yes | 1.49 | 0.194 | 1.109, 1.88 | |
| **Contrast** | **Log odds ratio** | **SE** | **df** | **95% CI** |
| No - Yes | -0.266 | 0.224 | 177 | -0.708, 0.176 |
| **Location** | | | | |
| **Level** | **Estimated marginal mean** | **SE** | **95% CI** | |
| Dundee | 0.976 | 0.178 | 0.625, 1.33 | |
| Edinburgh | 1.638 | 0.192 | 1.259, 2.02 | |
| Hull | 0.918 | 0.194 | 0.536, 1.30 | |
| USA | 1.620 | 0.197 | 1.232, 2.01 | |
| **Contrast** | **Log odds ratio** | **SE** | **df** | **95% CI** |
| Dundee - Edin | -0.6619 | 0.258 | 175 | -1.172, -0.152 |
| Dundee - Hull | 0.0581 | 0.261 | 175 | -0.457, 0.573 |
| Dundee - USA | -0.6441 | 0.262 | 175 | -1.161, -0.128 |
| Edin - Hull | 0.7200 | 0.270 | 175 | 0.188, 1.252 |
| Edin - USA | 0.0177 | 0.270 | 175 | -0.515, 0.550 |
| Hull - USA | -0.7023 | 0.273 | 175 | -1.241, -0.164 |
