# Supplementary material for: Anatomy Nights: An international public engagement event increases audience knowledge of brain anatomy
Source: PLoS One. 2022 Jun 9;17(6):e0267550. doi: 10.1371/journal.pone.0267550 (PMC9182231; doi:10.1371/journal.pone.0267550)
Supplement: Attachment — Submitted filename: Response to Reviewers.docx [file pone.0267550.s006.docx]

Reviewer #1: Abstract

No research methods, design, and data collection instrument were mentioned in the abstract

The abstract now reflects the research design better than before. We hope the reviewer finds this acceptable.

Introduction
Avoid usage of long sentences, particularly in paragraph 2. Improve referencing, for instance sentence beginning with “stroke….lacking”; requires reference. Line 75 -78
Line 90 ending with countries hosting Anatomy Nights: It will be interesting to expand the statement by pointing out that not all countries host Anatomy Nights and stating regions which commonly host such Anatomy Nights predominantly.
It will also be interesting in the background before giving the aim of the study, to outline if the Anatomy Nights are common in your study setting and the usual attendance.

Thank you for these comments, as they helped us set up our introduction to Anatomy Night much better. To be clear, these events are not common in our field and were designed precisely because they are not common and are an effort to bring anatomy education back into public spaces, consistent with the history of the discipline but not in heavy practice since the Victorian era. We have made edits to the introduction to reflect this set up better.

Materials and methods
Outline clearly research approach and design applicable in the study. Outline target population and calculation of sample size. As alluded in the results section that participants amounted to 102, how did you arrive at it? Moreover, outline the sampling method clearly, how did sample the 102 persons. Did you sample every person attending Anatomy Nights? If so, were all in attendance over the age to consent? You also did not highlight use of informed consent. How was sample calculated, which technique was used, including how was sampling down. Did you sample every participant coming in. Include clear data analysis method used. Specify specific statistical test used.

Thank you for pointing where we had unclear descriptions of the work and for identifying where we could better demonstrate effect sizes for our results. Everyone in attendance at Anatomy Night events was invited to participate in the pre- and post-event survey research. Participants were provided “participant information sheets” detailing the purpose of the quiz and how their quiz submission would be used. The inclusion of participants under the age of 16 was covered in our ethics application and approval, and it should be noted that due to the venues used for Anatomy Nights, anyone under the age of 16 would have been accompanied by an appropriate adult (also note that only 2 participants in this study were aged 16 years or under). This research, as detailed in the manuscript, was designed to assess the baseline knowledge of our participants and whether anatomical knowledge was gained from participation in Anatomy Night. We have been very explicit now about which statistical approaches we used, and how we interpret our results. Our materials and methods section has been revised to reflect this:

“We asked two general questions of the data. First, do scores differ between testing pre- vs. post- the Anatomy Nights talks and dissection? We addressed this question using a generalized linear model (R’s glm function; R version 4.1.0 [21]) that assumed a binomial distribution and implemented a logit link function. Here, and throughout, we present back-transformed (generalized) means, standard errors and 95% confidence intervals (hereafter, 95% CI) for each treatment level. We present the magnitude of the effect of pre- vs. post- testing on scores as an odds ratio, (with strandard error and 95% CI). calculated with R’s ‘emmeans’ package (V 1.6.1). Throughout, odds ratio estimates (and associated error) are also based on generalized means.

Second, we investigated whether the participants’ Academic Qualification (school, undergraduate, postgraduate, none), employment in the healthcare sector (Yes, No), and location (Dundee, Hull, Edinburgh, Indianapolis) affected test scores. Specifically, we tested whether each of these factors affected *i)* the magnitude of the *change in score* between pre- and post- sessions (i.e., quantified by an interaction term between test timing and the focal factor), and *ii)* the average score attained. We implemented three models for each of these three factors (i.e., education, employment type, location). Model type ***(a)*** included a term for the test timing (pre- vs. post-), a term for the given factor (e.g., location), and a term accounting for the interaction between the given factor and timing; model type ***(b)*** was identical to type *(a)*, but lacked the interaction term; model type ***(c)*** included a term for timing, only. We compared models using likelihood ratio tests to determine whether a focal factor influenced test scores. For example, for a given factor type (e.g., employment type), we compared model type *(a)* vs. *(b)* to determine whether the interaction term affected test scores and *(b)* vs. *(c)* to test whether the focal factor affected average scores. Note that two participants had no Academic Qualification (category ‘none’). We excluded these two participants from the analysis of Academic Qualification because the sample size for this group (‘none’) was too small to effectively compare it to the remaining three Academic Qualification categories.

We using the glmmTMB function [22] (Version 1.1.2) to model test performance with generalized mixed-effects models, implementing a binomial response distribution and logit-link function, Models included the fixed effects terms described, above (models *a* - *c*), Participant ID as a random effect, and an second random effect (‘units’) that modeled overdispersion.

As described in Results, likelihood ratio tests revealed little evidence for interactions between test timing and each of the three focal factors (Academic Qualification, Employment type, Location). Therefore, we calculated mean test performance (averaging over test timing) and effect size for each level of each focal factor using models that lack an interaction (i.e., model type (b)). Appendix Table A1 presents these estimates and effect sizes (again) on the latent scales.

We used binomial tests to determine whether the probability of correctly answering the question regarding stroke differed significantly from the random expectation of 25%; ‘pre-‘ and ‘post-’ data were analyzed separately.

We do not test whether performance changed between ‘pre-‘ and ‘post-‘ time periods for individual questions because exceedingly high test performance led to poor performance by Generalized Linear Models. In particular, all participants answered Question 3 correctly post-lecture and dissection (i.e., 93 / 93 answered correctly), which led to nonsensical effect size estimates (and 95% CI’s) for this question. Therefore, we report results for individual questions descriptively.

All data and R scripts are available (S2-3 Files) to allow readers to replicate our analyses.”

Results
Demographic section,
Did you select every person who attended the Anatomy Nights, if so, this should be clear in the method subsection? Explain how you arrived at 102. Furthermore, as you indicated that nine participants did not fully complete the questionnaire/sheets, you should explain in your manuscript if you did consider sampling for attrition rates to cover for incomplete sheets. Although you did not. Revise accordingly.

Our results section has been rewritten to reflect the changes described in our revised materials and methods section. See response above

anatomy knowledge
I suggest the inclusion of table showing pre-and-post outcomes on specific questions

We detail the outcomes of pre- and post-survey results for specific questions in the results and discussion sections.

Discussions and conclusion
No comments, except to say well discussed and concluded.

Thank you for the positive feedback!

Overall comments
Avoid use of long sentences. Revise, proofread and edit.

Thank you.

Recommendation for approval

The manuscript can be revised with consideration of the comments and re-submitted for approval.
