# Supplementary material for: Anatomy Nights: An international public engagement event increases audience knowledge of brain anatomy
Source: PLoS One. 2022 Jun 9;17(6):e0267550. doi: 10.1371/journal.pone.0267550 (PMC9182231; doi:10.1371/journal.pone.0267550)
Supplement: Attachment — Submitted filename: PLOS One Anatomy Night response to reviewers 11.16.21.docx [file pone.0267550.s007.docx]

16 November 2021

Ebrahim Shokoohi

Academic Editor, PLOS One

Re: PONE-D-21-16218R1

Dear Dr. Shokoohi,

Thank you for the review of our revised manuscript. We are grateful to the reviewers for pointing out where we could strengthen our writing to be clearer. We have addressed each of the reviewers’ comments, and our responses are indicated in red font below. We hope this revision satisfies the journal and we look forward to a positive decision soon on this manuscript.

Best wishes,

Kat Sanders

Reviewer #1: The previous comments were partially addressed.
Abstract
Mention in the abstract research methods, design and data collection instrument applied in the study.

We have reworded to the abstract to better reflect the research methods, design, and data collection instruments in the study.

Method
How was sampling conducted?
be specific also on population, methods and design.

Our sampling was described in the previous revision of the manuscript. However, the reviewer’s comment prompted us to re-evaluate how clear our description was. To that end, we have rearranged the first paragraph of the Data Collection section to be clearer. Our sampling strategy is clearly described in lines 148-162. Furthermore, we provide the pre- and post-test surveys as a supplemental file (S1) for complete transparency.

It would be interesting to identify the type of harm which could occur and how to measurers to address it.

Whereas the reviewer finds this to be interesting, it is not within the scope of the current research design or the scope of the manuscript. We thank the reviewer for the comment but have chosen not to address this comment because it does not work in service to the research as presented.

Also you indicated that children under the age of 16 were accompanied by guardians or parents, but it is confusing as to whether did they consent on their behalf.The statement may need to be reworded.

As described above, we reworded this statement to clearly state that guardians/parents consented on behalf of their respective minors by submission of the survey itself. Participants were informed of this procedure for obtaining informed consent at the top of each survey page.

Implement the major methodological recommendation for approval for publication

Reviewer #2: Dear Authors
I have checked the revised manuscript submitted. Despite the questions raised and addressed by the Authors, the statistical analysis need to be clarified in the MM. How the statistical performed?

We respectfully disagree with the reviewer that the statistical analysis needs to be clarified further. In lines 206-260, we have clearly described all of the statistical tests used as well as which functions in R were used. Furthermore, as we did with the last revision, we provide supplementary files that include all the raw data from the surveys (S3), all estimates from generalized linear mixed models (S2), and the R scripts used to analyze the data (S4) so that anyone interested can re-run the analyses if they wish. This is excellent practice, exemplifies transparency, and we could not be more detailed in this manuscript.
