# Supplementary material for: Anatomy Nights: An international public engagement event increases audience knowledge of brain anatomy
Source: PLoS One. 2022 Jun 9;17(6):e0267550. doi: 10.1371/journal.pone.0267550 (PMC9182231; doi:10.1371/journal.pone.0267550)
Supplement: Attachment — Submitted filename: Response to reviewers v2.docx [file pone.0267550.s009.docx]

We have not included a response to reviewers, as the editor’s comments stated that only their minor edits and change to affiliations was required. We believe this is likely because the only review comment came from Reviewer 1 and we had already addressed that comment in a previous submission.

In response to the evaluation of the manuscript, we have added to the following sentence on page 7 lines 157-8:

This process of gaining informed consent from participants aged 16 years and over was explicit in the ethics applications to the institutional committees, **who thus waived the need for parental concern for participants aged 16 and 17 years old.**

We hope this provides the necessary clarity.

Editor’s comments 16/12/21

Dear Authors

I have checked the submitted revised manuscript. All the questions raised by the Referees were addressed. However, only some minor changes to the affiliation, which yellow highlighted, are needed. The text corrections also need to be considered. Please check the attached file. After the minor changes done, it can be accepted for publication.

Regards,

Ebrahim Shokoohi (Academic Editor)
